# Young people's use of e-cigarettes in Wales, England and Scotland before and after introduction of EU Tobacco Products Directive regulations: a mixed-method natural experimental evaluation

**DOI:** 10.1016/j.drugpo.2020.102795

**Published:** 2020-11

**Authors:** Graham Moore, Rachel Brown, Nicholas Page, Britt Hallingberg, Olivia Maynard, Jennifer McKell, Linsay Gray, Anna Blackwell, Emily Lowthian, Marcus Munafò, Anne-Marie Mackintosh, Linda Bauld

**Affiliations:** a Centre for the Development and Evaluation of Complex Interventions for Public Health Improvement, Cardiff University; b Usher Institute of Population Health Sciences & Informatics, University of Edinburgh; c Institute for Social Marketing, University of Stirling and UK Centre for Tobacco and Alcohol Studies; d MRC Integrative Epidemiology Unit (IEU) at the University of Bristol, Bristol, UK/UK Centre for Tobacco and Alcohol Studies (UKCTAS) and School of Psychological Science, University of Bristol, Bristol, UK; e MRC/CSO Social and Public Health Sciences Unit, University of Glasgow; f SPECTRUM Consortium, UK; g Cardiff School of Sport & Health Sciences, Cardiff Metropolitan University, Wales, UK

**Keywords:** E-cigarette, Policy, Adolescence, Tobacco, Mixed methods, Interrupted time series analysis

## Abstract

**Background:**

Young people's experimentation with e-cigarettes has increased in recent years, although regular use remains limited. EU Tobacco Products Directive (TPD) regulations introduced packet warnings, advertising restrictions, and regulated nicotine strength from 2016, in part due to concerns regarding use by young people. This paper examines e-cigarette use trajectories before and after TPD.

**Methods:**

E-cigarette use data were obtained from School Health Research Network/Health Behaviour in School-aged Children surveys in Wales and Smoking Drinking and Drug Use surveys in England. Data from Wales were analysed using segmented logistic regression, with before and after regression analyses of English data. Semi-structured group interviews included young people aged 14-16 years in Wales, England and Scotland in 2017 and 2018.

**Results:**

In Wales, ever use of e-cigarettes increased over time, but under a range of assumptions, growth did not appear to continue post-TPD. A small and non-significant change in trend was observed post-implementation (OR=0.96; 95%CI=0.91 to 1.01), which increased in size and significance after adjusting for ever smoking (OR=0.93; 95%CI=0.88 to 0.98). There was little increase in regular e-cigarette use from 2015 to 2017 in Wales. However, ever and regular use increased from 2014 to 2016 in England. Young people in all nations described limited interactions with components of TPD, while describing e-cigarette use as a ‘fad’, which had begun to run its course.

**Conclusions:**

This study provides preliminary evidence that young people's e-cigarette experimentation may be plateauing in UK nations. The extent to which this arises from regulatory changes, or due to a fad having begun to lose its appeal among young people in the UK countries, remains unclear. These trends contrast to those observed in North America, where newer products whose EU market entry and marketing have been impacted by TPD, have gained traction among young people. Long-term monitoring of e-cigarette use trends and perceptions among young people remain vital.

## Background

E-cigarettes emerged in United Kingdom nations from around 2011 (West, Beard, & Brown, 2018), with use primarily among smokers and ex-smokers (McNeill, Brose, Calder, Bauld, & Robson, 2018). Harms remain disputed, and there are divergent perspectives among policymakers, health professionals and the public on how to manage uncertainty (Romijnders, van Osch, de Vries, & Talhout, 2018; Stepney, Aveyard, & Begh, 2019). However, in a recent randomised trial, e-cigarettes were almost twice as effective in supporting smoking cessation as other nicotine replacement therapies, where both arms also received behavioural support (Hajek, et al., 2019). Given growing evidence of impacts on cessation (Bullen, et al., 2013; West, Shahab, & Brown, 2016), and good reason to believe e-cigarettes are safer than smoking, many endorse use within harm reduction strategies (McNeil, et al., 2015).

A key concern driving regulation of e-cigarettes has however been their perceived role as a ‘gateway’ into smoking for young people, or perceptions that they may renormalize smoking (Chapman, Bareham, & Maziak, 2018; Conner, et al., 2018). There is growing longitudinal evidence that e-cigarette use among never smoking young people is associated with higher risk of taking up smoking (Glasser, Abudayyeh, Cantrell, & Niaura, 2018), although causality is contested, and tobacco and e-cigarettes may be driven by shared risk factors. While often conflated with ‘gateway’ in public discourse (Thompson, 2019), theories of renormalisation (Voigt, 2015) focus on sociological processes through which presence of e-cigarettes in society influences perceptions of smoking. According to the renormalisation hypothesis, growing visibility of e-cigarette use may increase acceptance of the ‘similar’ behaviour of smoking, and hence, smoking uptake. To date, there is little evidence that this is occurring in the UK. Analyses of survey data from three UK nations found that experimentation with tobacco declined during the emergence of e-cigarettes, while the decline in acceptability of smoking accelerated (Hallingberg, et al., 2018). Similar findings have been reported among older adolescents and young adults in the United States (US) (Levy, et al., 2018). In more recent UK surveys, the secular decline in smoking has begun to level off, mirroring trends across a broad range of adolescent risk behaviours (Hewitt, et al 2019; NHS Digital, 2018).

Concerns regarding young people's use of e-cigarettes intensified following a recent outbreak of pulmonary conditions and links to use of e-cigarettes in the US (Hammond, 2019). While this situation is evolving, the latest evidence indicates that these incidents are largely related to chemicals which are not present within products sold legally in the UK (Alexander & Perez, 2019; Nyakutsikwa, Britton, Bogdanovica, & Langley, 2019). Nevertheless, there remains consensus that use of e-cigarettes by young people ought to be prevented, regardless of whether or not they cause smoking. Young people's use of e-cigarettes was first measured from 2013 in Wales and Scotland, and 2014 in England. Experimental use of e-cigarettes has become more prevalent than experimentation with tobacco (de Lacy, Fletcher, Hewitt, Murphy, & Moore, 2017; Moore, et al., 2015), though regular use has to date been limited, primarily occurring among smokers (Bauld, et al., 2017). In the US, by contrast, rapid growth in use of newer generation e-cigarette products by young people has been reported (Kavuluru, Han, & Hahn, 2019).

**Figure 1:**
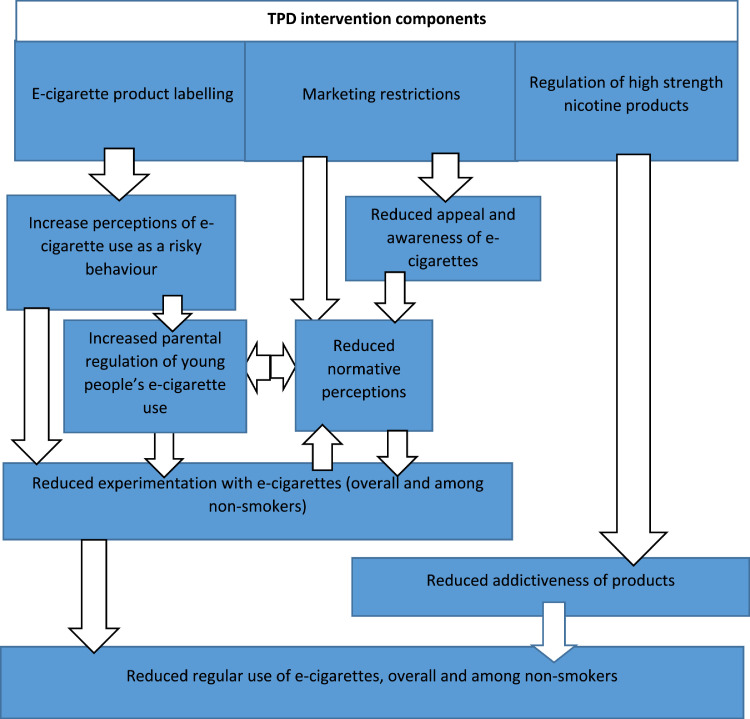
Logic model for potential effects and mechanisms of TPD e-cigarette regulation

Public health communities therefore face a challenging balance of regulating sufficiently to prevent harms to young people, while not making e-cigarettes unattractive or inaccessible as an alternative for smokers. Internationally, regulations introduced to reduce young people's e-cigarette exposure include age of sale restrictions (Dutra, Glantz, Arrazola, & King, 2018). From May 2016, the European Union (EU) introduced a range of further regulations. Tobacco Products Directive (TPD) regulations (see Figure 1) imposed mandatory warnings across 30% of the packet surface for e-cigarettes containing nicotine, banned many forms of advertising, and introduced restrictions on nicotine strength of non-medicinal products (Official Journal of the European Union, 2014). Throughout the UK, regulation co-occurred with additional regulation of tobacco, including plain packaging (Breton, Britton, Huang, & Bogdanovica, 2018). Marketing restrictions came into force immediately, while unregulated products were phased out over a 12-month period and could no longer be sold from May 2017.

Evidence from tobacco research indicates that prominent health warnings can influence risk perceptions, with size of warning equated to scale of risk (Hammond, 2011). Health warnings on e-cigarette packages may increase the extent to which they are viewed as risky. However, there is evidence that e-cigarettes are already viewed as risky by young people (Berg, et al., 2015; Weishaar, Trevisan, & Hilton, 2016), particularly non-users (Bernat, Gasquet, Wilson, Porter, & Choi, 2018). Further, experimental evidence suggests TPD health warnings may have the unintended consequence of making e-cigarettes less appealing to smokers (Cox, Frings, Ahmed, & Dawkins, 2018). During emergence in the UK, many argued that marketing for e-cigarettes echoed tobacco industry marketing and was directed toward young people (Bauld, Angus, & De, 2014; McCarthy, 2014). In the US, young people's exposure to television advertising for e-cigarettes increased almost three-fold from 2011 to 2013 (Duke, et al., 2014) with links between exposure to advertising and use (Mantey, Cooper, Clendennen, Pasch, & Perry, 2016). While removal of many forms of marketing might plausibly impact e-cigarette use, online marketing (Singh, et al., 2016) and point of sale displays (Best, et al., 2016) which have both been associated with use, are unregulated. The nicotine concentration of non-medicinally regulated e-cigarette devices is controlled within TPD, and emerging high concentration products gaining traction among adolescents elsewhere (Kavuluru, et al., 2019) have not been able to enter EU markets such as the UK as easily, largely due to these regulations.

While the manner of the UK's planned 2020 departure from the EU following the 2016 Brexit vote remains unclear, the English government has committed to a review of TPD regulations by May 2021 (Department for Health and Social Care, 2016). Hence, evaluation of its effects on young people is critical. Estimating effects of such legislation relies on epidemiological methods which are subject to bias (Craig, et al., 2012). For emerging public health issues such as e-cigarette use, challenges are amplified by the data poor environment, including availability of a short time series by which to understand secular baseline trends prior to regulation. Combining outcomes data with process evaluation data (Moore, et al., 2015) becomes critical in evaluating such moves; quantitative data allow estimation of changes in use post-intervention, while qualitative data enable identification of competing and complementary causal hypotheses for why any change may or may not have occurred. This paper draws upon surveys in Wales and England to examine changes in trends in e-cigarette use after the introduction of TPD regulations. Trends are interpreted through use of qualitative interviews with secondary school-aged young people in England, Wales and Scotland, collected during the transitional period of TPD implementation, and 12-months later. In combination, these data address the following research questions:

• RQ1: Did the level and/or rate of growth for young people's e-cigarette experimentation change significantly after introduction of TPD regulations?
• RQ2: How did young people perceive and interact with the components of TPD regulations over time?
• RQ3: What complementary and competing causal hypotheses are offered via qualitative data for estimated change (or lack of change) in trends for young people's vaping?

## Methods

### Study design

The study adopted a parallel mixed-method research design; quantitative and qualitative data were collected simultaneously and analysed separately prior to integration. Quantitative data were from the 2013, 2015 and 2017 School Health Research Network/Health Behaviour in School-aged Children (SHRN/HBSC) surveys in Wales, and the 2014 and 2016 Smoking Drinking and Drug Use (SDDU) surveys in England (NatCen Social Research, 2015). Further details of individual datasets are reported elsewhere (Hewitt, et al 2019; NHS Digital, 2018). Qualitative data were from group semi-structured interviews with 14-15 year olds in England, Scotland and Wales conducted from March to June 2017 and repeated (where possible, with the same young people, supplemented by additional participants) from February to July 2018, providing insights into perceptions of e-cigarettes beyond the quantitative time series.

### Quantitative study component - survey data sources

Measures of e-cigarette use were introduced to HBSC Wales in 2013. A SHRN survey was conducted in 2015 modelled on HBSC, with HBSC Wales nested into the SHRN survey in 2017. SDDU introduced measures of e-cigarette use in a nationally representative cross-sectional sample in England from 2014. Surveys are conducted with 11-16 year olds in secondary schools. In order to harmonise age ranges across nations included in our study (long-term analyses will integrate Scottish data only collected from 13 and 15 year olds), our study focused on young people in Years 9 and 11 (i.e. aged approximately 13 and 15 years).

#### Measures

In all surveys, pupils are asked to indicate their sex. Year group is used in SHRN/HBSC as a proxy for age, whereas in SDDU pupils are banded by age. Pupils are asked to indicate their ethnicity, with participants categorised as either ‘White’ or ‘Black and Minority Ethnicity’ (BME). Socioeconomic status (SES) is assessed in Wales using the Family Affluence Scale (Hartley, Levin, & Currie, 2016). No consistent SES measure was available for SDDU over the time series. In SHRN/HBSC, e-cigarette use is measured using two questions, one for ever use (Have you ever used an e-cigarette?) and in 2015/2017 only, one for current use (“How often do you use an e-cigarette at present?’). In SDDU, a single item is used to derive ever use and use weekly or more: pupils are instructed to read the following statements and select which one describes them; ‘I have never tried electronic cigarettes’, ‘I have used electronic cigarettes only once or twice’, ‘I used to use electronic cigarettes but I don't now’, ‘I sometimes use electronic cigarettes, but I don't use them every week’ and ‘I use electronic cigarettes regularly, once a week or more’. In SHRN/HBSC, smoking status is measured using two questions, one for ever use derived from age of first use (“At what age did you do the following things? – smoke a cigarette (more than a puff)” – coded as never vs all other) and another for current regular use (“How often do you smoke tobacco at present?”). In SDDU, smoking status is derived using a similar single item question, with equivalent response options to that applied to e-cigarettes.

#### Statistical analysis

Our primary analysis takes the form of an interrupted time series (ITS) design (Bernal, Cummins, & Gasparrini, 2016). We compared survey data before and after introduction of the regulations (May 2016) to estimate deviation in e-cigarette use from the baseline trend. Each Welsh survey can be broken down into 3 or 4 monthly time-points, to facilitate segmented regression analysis. English data do not offer this capability and hence provide only one time-point for each pre and post TPD period. For Welsh data, we use logistic regression to model the binary outcome ever e-cigarette use. The basic model for a segmented regression analysis, modelling both change to slope and level, is given by:

$$logit\left(Y_{ki}\right)=\beta_{0}+\beta_{1}{\left(time\right)}_{ki}++\beta_{2}{\left(intervention\right)}_{ki}++\beta_{3}{\left(postslope\right)}_{ki}+\varepsilon_{ki}$$

where Y_ki_ is the e-cigarette use status outcome of individual *i* at time *k; time* is a continuous variable indicating time (in months) from the start of the study up to the end of the period of observation; *intervention* is coded 0 for pre-intervention time points and 1 for post-intervention time points and *postslope* is coded 0 up to the last point before the intervention phase and coded sequentially from 1,2,… thereafter (Lagarde, 2011). *β*_0_ estimates the baseline level of e-cigarette use at time 0 (beginning of the period); *β*_1_ estimates the structural trend in e-cigarette use independently from TPD implementation; *β*_2_ estimates the immediate impact of TPD implementation or the change in level in e-cigarette use after TPD implementation; and *β*_3_ estimates the change in trend in e-cigarette use, after intervention. As TPD was introduced over a 12-month period, we anticipated that change in trend, rather than sudden change in level at the intervention point, was more likely. Hence, interpretation notes evidence of change in level, but focuses predominantly on change in trend. As a post-hoc sensitivity analysis, we included quadratic terms for time to account for non-linearity (see Supplementary Material). Our secondary outcome of regular vaping was available for 2015 and 2017 in Wales, and analysed using a before and after binary logistic regression comparison. Likewise, for both outcomes in English data, binary logistic regression analyses were performed. For, Welsh data standard errors were inflated to account for school-level clustering, although this was not possible for English data due to removal of school identifiers by the data owners. Analyses are presented for all pupils, and stratified by age, sex, ethnicity, smoking status and (Wales only) SES. In the absence of a non-intervention comparator, causal inference may be strengthened if changes in outcomes of interest are not observed for another unrelated risk behaviour (Bonell, et al., 2011; Cousens, et al., 2011). Hence, we test whether interruption to time series in e-cigarette use is paralleled by similar interruption in energy drink consumption (selected principally because use was unlikely to be impacted by introduction of TPD regulations, and did not follow the secular decline observed for most other substances). All analyses of change over time, including sub-group analyses, were agreed with our independent Study Steering Committee (SSC) prior to analysis taking place, unless described as post-hoc analyses to investigate emerging issues. All analyses were presented to our SSC prior to submission. In interpreting estimates of change we are aware of debates regarding over-attention to statistical significance (McShane, Gal, Gelman, Robert, & Tackett, 2019), and counter-arguments that privileging point estimates risks ignoring variation in precision (Brown, et al., 2019). Hence, we compare and contrast models on the size of ‘effect’ observed, while presenting 95% confidence intervals to indicate where a pre-determined threshold for determining that between group difference is present has been achieved.

### Process evaluation

#### Semi structured pupil interviews

##### Recruitment and sampling

We sampled schools from Wales, England and Scotland purposively in order to achieve a varied sample in terms of country, SES (i.e. free school meal entitlement), and urbanisation (Brown, et al., 2020). Within each school we conducted up to 4 group-based interviews with 2-6 young people recruited for each. While smoking rates have historically been higher among girls, with recent convergence, the opposite is true of e-cigarettes, which are more popular among boys (Hewitt, et al 2019; NHS Digital, 2018). Hence, we conducted single-sex group interviews and asked school staff to identify pupils from a range of higher and lower ability classes. To maximise rapport and interaction among young people, friendship groups were identified by school staff. As we were interested in perceptions regardless of smoking or vaping status, we did not explicitly attempt to recruit tobacco smokers or e-cigarette users, and advised teachers of this. Interviews were held on school premises, with one or two researchers facilitating each interview. While it was not possible to interview young people prior to legislation, we aimed to interview young people as early as possible before the date of full compliance, and again almost one year later. Our aim was to recruit 12 schools (4 per country). However, contracting and ethical approvals were not in place until March 2017, limiting recruitment to 7 schools for baseline interviews. We followed the same young people within these schools for longitudinal interviews, and recruited 4 additional schools to provide a greater breadth of data after full implementation in 2018. Distinct datasets were generated, one incorporating data from pupils interviewed at two time points (2017 and 2018, n=62, interviewed in Year 10/S3 and again in Year 11/S4) and another from those interviewed only in 2018 (n=86, a mixed sample of interviewees mostly from Year 10/S3 and a smaller number from Year 11/S4). Data on all those interviewed in 2018 were also analysed in relation to topics that were not associated with the passage of time, e.g. observations of new warning labels on e-cigarettes after the full compliance date.

##### Interview schedules

Informed by our logic model (Figure 1), interviews explored: i) perceptions of e-cigarettes, tobacco and the inter-relationship between the two; and ii) how these perceptions were impacted by key elements of TPD regulation, such as product labelling and marketing restrictions. Interviews explored the broader context in which this legislation is implemented, including simultaneously introduced regulation to make tobacco less appealing and accessible to young people. Groups were encouraged to express and explore disagreements and share understandings, with divergence of views indicated in the results.

##### Analysis

Transcripts were initially analysed and then second-coded to facilitate exploration of distinct themes and compared after initial analysis to identify areas of convergence. Verbatim quotes are used throughout to illustrate key themes and pupils are identified by country code (W, S, E), school code, group number and sex (e.g. E1(3)F = country E, school 1, group 3, girl). Reference is made to the number of groups in which a specific theme was discussed but this refers to frequency of emergence rather than uniformity of opinion.

##### Quantitative process indicators (SHRN 2017 only)

A number of quantitative process indicators were included in the 2017 SHRN survey, providing a cross-sectional snapshot mapping onto components of TPD and mechanisms within our logic model (Figure 1). Measures include: i) relative risk perceptions for tobacco and e-cigarettes; ii) perceived parental approval of tobacco and e-cigarettes; iii) exposure to e-cigarette marketing; and iv) content of e-cigarettes used by young people. Informed by emerging themes from our qualitative data, we included survey items on where e-cigarettes were obtained from. These indicators are presented overall, and by demographic variables and smoking status, and integrated within the qualitative data.

#### Data integration

Statistical analyses were undertaken by BH and NP, with guidance from LG. Qualitative data were analysed by RB and JM naïve to quantitative outcomes. GM subsequently integrated themes from quantitative and qualitative data in consultation with RB and NP. Integration focused on areas of agreement, disagreement and elaboration across data sources.

#### Ethics and consent

Ethical approval was provided by the Cardiff University School of Social Sciences Research Ethics Committee. Quantitative analyses involved secondary analyses of anonymised secondary data, held by the authors or accessed via the UK Data Archive. Consent for pupil interviews comprised three stages: school (also local authority in Scotland); parent (letters for parents describing the study with a consent form to return if they were happy for their child to participate); and pupil. While our ethics committee required opt-in parental consent for the first round of interviews, for schools recruited in 2018, this was reversed, and a more standard opt-out process approved.

## Results

### Quantitative components

#### Ever e-cigarette use - Wales

Data were available for 12 time points between November 2013 and December 2017, representing 51,056 young people in Wales. Prevalence of ever use of e-cigarettes almost doubled over the time series: 17.6% of pupils sampled reported ever use of e-cigarettes in November 2013 compared to 34.0% in December 2017 (Supplementary Table 1).

**Figure 2:**
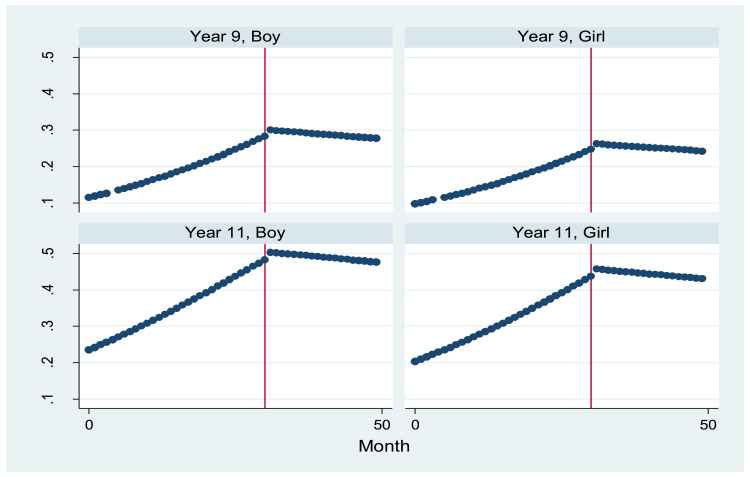
Predicted probabilities for ever use of e-cigarettes by year group and gender for period November 2013 to December 2017 (intervention point: May 2016)

For the whole sample and across age and gender sub-groups, estimates of time trend indicated an approximately 4% (i.e ORs=1.04) increase in odds of ever use per month (see Table 1). There was no clear evidence, under any assumptions, of a step-change in prevalence (indicated by the variable ‘level’; see also Supplementary Table 2), with direction and size of odds ratios varying substantially between sub-groups, and confidence intervals wide. However, for the whole sample and all subgroups, odds ratios for post-slope were in the anticipated direction of negative change in trend, indicating an approximately 4% reduction in odds of ever e-cigarette use each month beyond the TPD implementation date, relative to the secular baseline trend. Reduction in growth of ever use was greater for boys than girls, with an estimated 6% reduction in odds per month for boys vs 2% for girls. As depicted graphically in Figure 2, segmented regression models suggested no further growth in ever use post TPD implementation.

**Table 1:** Odds ratios (and 95% CIs) for ever use of e-cigarettes among pupils in Wales between 2013 and 2017, overall and by gender, school year, and smoking status (segmented regression analyses)

			*P*
All (n=51,056)	Time (month-year)	1.04 [1.03, 1.05]	<0.001
	Time^2^	-	-
	Level	1.09 [0.41, 2.89]	0.860
	Post-slope	0.96 [0.91, 1.01]	0.125
Boys (n=24,993)	Time	1.04 [1.03, 1.05]	<0.001
	Time^2^	-	-
	Level	1.88 [0.70, 5.06]	0.213
	Post-slope	0.94 [0.89, 0.99]	0.020
Girls (n=26,063)	Time	1.04 [1.03, 1.05]	<0.001
	Time^2^	-	
	Level	0.64 [0.19, 2.22]	0.485
	Post-slope	0.98 [0.91, 1.05]	0.587
Year 9 (n=28,471)	Time	1.04 [1.02, 1.05]	<0.001
	Time^2^	-	-
	Level	1.49 [0.39, 5.68]	0.563
	Post-slope	0.94 [0.88, 1.02]	0.124
Year 11 (n=22,585)	Time	1.04 [1.03, 1.05]	<0.001
	Time^2^	-	
	Level	0.85 [0.26, 2.78]	0.789
	Post-slope	0.97 [0.91, 1.04]	0.375
Never smoker (n=40,703)	Time	1.06 [1.04, 1.07]	<0.001
	Time^2^	-	-
	Level	1.05 [0.39, 2.83]	0.917
	Post-slope	0.94 [0.89, 0.99]	0.022
Ever smoker (n=8,746)	Time	1.07 [1.06, 1.08]	<0.001
	Time^2^	-	-
	Level	2.34 [0.36, 15.38]	0.376
	Post-slope	0.89 [0.80, 0.98]	0.024

Note: where appropriate, models adjusted for gender and/or school year

For never and ever smokers, the time trend in ever use of e-cigarettes was steeper than for the whole sample, with an estimated 6% and 7% increase in odds of ever use per month respectively (Table 1). There was also suggestion of larger changes in trend for both groups than for the whole sample, with odds declining by 6% per month post-implementation for never smokers (OR: 0.94; 95% CIs: 0.89-0.99) and by 11% for ever smokers (OR: 0.89; 95% CIs: 0.80-0.98). As a post-hoc investigation to understand these differences, a binary term for ever smoking was added to the whole group model (Supplementary Table 3). Negative change in trend post-intervention increased to 7%, from 4%, and became statistically significant (OR: 0.93; 95% CIs: 0.88-0.98).

A quadratic term for time was itself not significant, but inclusion led to an increase in the odds ratio for the level and a decrease in the odds ratio for the post-slope, leading to non-significance of previously significant associations (Supplementary Table 4). As a further post-hoc analyses following comments of an anonymous reviewer, we harmonised the sample across years by estimating ever use by year for a subsample of schools (n=42) who participated in all 3 years, finding a similar pattern to the main analyses, with ever use almost doubling from 15.1 (95%CI=13.4 to 16.8%) in 2013 to 28.0% (26.7% to 29.2%) in 2015, but subsequently increasing by a smaller margin to 35.1% (34.1 to 36.1%).

#### Regular e-cigarette use in Wales

Data on regular e-cigarette use were available for 2015 and 2017 (n=47,266). Prevalence increased marginally: 4.2% of pupils reported regular e-cigarette use in 2015 compared to 4.8% in 2017 (Supplementary Table 5). Results of before and after models are presented in Table 2. Odds ratios indicate an estimated 7% increase in the odds of regular e-cigarette use between 2015 and 2017, falling short of statistical significance (OR: 1.07; 95% CIs: 0.98-1.17). Across sub-groups, odds ratios were indicative of small increases in odds of regular vaping, with negligible increases for girls, older students, and regular smokers, though with increases in odds of approximately 14% (i.e. ORs=1.14) among younger (Year 9) pupils (p=0.04) and never smokers (p=0.08). Additional subgroup analyses provided evidence of marginally significant growth in regular e-cigarette use among White (OR: 1.09; 95% CIs: 1.00-1.20) and higher SES (OR: 1.13; 95% CIs: 1.02-1.25) pupils (Supplementary Table 6).

**Table 2:** Odds ratios for regular use of e-cigarettes among pupils in Wales between 2015 and 2017, overall and by gender, school year, and smoking status (before and after analyses)

			*P*
All (n=47,318)	Time	1.07 [0.98, 1.17]	0.118
Boys (n=23,095)	Time	1.11 [1.00, 1.24]	0.056
Girls (n=24,233)	Time	1.02 [0.91, 1.14]	0.770
Year 9 (n=26,390)	Time	1.14 [1.00, 1.28]	0.043
Year 11 (n=20,928)	Time	1.03 [0.93, 1.14]	0.571
Never smoker (n=38,014)	Time	1.14 [0.98, 1.31]	0.083
Ever smoker (n=7,920)	Time	1.03 [0.94, 1.12]	0.527

Note: where appropriate, models adjusted for gender and/or school year

#### Before and after analysis in England

In England, 8,178 pupils responded to questions on e-cigarettes. Increased prevalence of both outcomes were observed from 2014 to 2016: the proportion reporting having ever used e-cigarettes increased from 27.3% in 2014 to 32.5% in 2016, whilst regular use increased from 1.7% to 3.4% (Supplementary Table 7). Modelled changes in ever and regular e-cigarette use overall and by gender, age and smoking status, are presented in Table 4. These indicate increases overall between 2014 and 2016 for both ever and regular use outcomes, with odds of ever use increasing by 15% (OR: 1.15; 95% CIs: 1.10-1.22) and regular use by 47% (OR: 1.47; 95% CIs: 1.25-1.73). Increases were found across subsamples, with increases larger for boys than girls (see also Supplementary Table 8).

#### Falsifiability analysis – energy drink use in Wales

For energy drink use, there was no evidence of a time trend from 2013 to 2017. Changes in level and slope did not reach significance overall or for any subgroup (see Table 3 and Supplementary Table 10; and Supplementary Table 11 for quadratic models). However, for the whole sample, estimates of change in trend were of similar magnitude to those for e-cigarette use (4% decrease in the odds of use per month post-implementation). Unlike e-cigarettes, for which the inverse gender pattern was observed, change in trend for energy drink use was larger for girls, with an estimated 5% decline in odds of use compared to 2% for boys. For never and ever smokers, as for e-cigarette use, estimates of reduction in odds of energy drink use were slightly greater than estimated for the whole sample, as 5% and 6% respectively, though by a smaller margin than in the e-cigarette analysis.

**Table 3:** Odds ratios (and 95% CIs) for ever use of energy drinks among pupils in Wales between 2013 and 2017, overall and by gender, school year, and smoking status (segmented regression analyses)

			*P*
All (n=52,794)	Time (month-year)	1.00 [1.00, 1.01]	0.856
	Time^2^	-	
	Level	1.38 [0.60, 3.18]	0.453
	Post-slope	0.96 [0.92, 1.01]	0.089
Boys (n=25,888)	Time	1.00 [0.99, 1.00]	0.285
	Time^2^	-	
	Level	1.32 [0.55, 3.14]	0.534
	Post-slope	0.97 [0.93, 1.02]	0.272
Girls (n=26,906)	Time	1.00 [1.00, 1.01]	0.183
	Time^2^	-	
	Level	1.46 [0.51, 4.14]	0.477
	Post-slope	0.95 [0.89, 1.01]	0.075
Year 9 (n=29,545)	Time	1.00 [1.00, 1.01]	0.261
	Time^2^	-	
	Level	1.25 [0.44, 3.57]	0.673
	Post-slope	0.96 [0.90, 1.02]	0.150
Year 11 (n=23,249)	Time	1.00 [0.99, 1.00]	0.352
	Time^2^	-	
	Level	1.53 [0.55, 4.24]	0.415
	Post-slope	0.96 [0.91, 1.02]	0.199
Never smoker (n=41,457)	Time	1.00 [1.00, 1.01]	0.422
	Time^2^	-	
	Level	1.40 [0.60, 3.30]	0.437
	Post-slope	0.95 [0.91, 1.00]	0.054
Ever smoker (n=8,805)	Time	1.00 [0.99, 1.01]	0.794
	Time^2^	-	-
	Level	2.26 [0.63, 8.07]	0.210
	Post-slope	0.94 [0.87, 1.00]	0.065

Note: where appropriate, models adjusted for gender and/or school year

**Table 4:** Odds ratios (with 95% CIs) for primary and secondary outcomes among pupils in England between 2014 and 2016, overall and by gender, age, and smoking status

		Ever use of e-cigarettes		Regular use of e-cigarettes	
			*P*		*P*
All (n=8,178)	Time	1.15 [1.10, 1.22]	<0.001	1.47 [1.25, 1.73]	<0.001
Boys (n=3,994)	Time	1.18 [1.10, 1.27]	<0.001	1.64 [1.33, 2.03]	<0.001
Girls (n=4,184)	Time	1.12 [1.04, 1.21]	0.002	1.19 [0.91, 1.54]	0.204
13 year olds (n=3,987)	Time	1.14 [1.05, 1.24]	0.002	1.56 [1.16, 2.11]	0.004
15 year olds (n=4,191)	Time	1.16 [1.09, 1.24]	<0.001	1.43 [1.17, 1.73]	<0.001
Never smoker (n=6,160)	Time	1.16 [1.08, 1.25]	<0.001	1.55 [0.96, 2.52]	0.074
Ever smoker (n=1,911)	Time	1.32 [1.18, 1.47]	<0.001	1.52 [1.26, 1.82]	<0.001

Note: where appropriate, models adjusted for gender and/or age

Interaction terms for all models are presented in Supplementary Tables 12 and 13.

##### Process evaluation

Interviews with young people in England, Scotland and Wales and post-legislation quantitative indicators (Wales only)

#### Changes in perceived prevalence

This section first considers data from repeat qualitative interviews, outlining pupil perceptions of e-cigarette use among their peers, and changes from 2017 to 2018. This is then compared to 2018-only data to assess current views on prevalence.

In 2017, a large majority of pupils across all groups stated that e-cigarette use had risen rapidly among their age group and was, at that point, more common than tobacco smoking. For most, this was discussed in terms of being something that was ‘tried’ as a shared experience with friends rather than used regularly:

I think quite a few people try it. Not maybe … not consistently or the whole time, but just occasionally (E1(3)F)

They just like, hang about with the crowd. And just use e-cigarettes because that's what the rest of them are doing. (S1(2)F)

This casual, or experimental, use was widely accepted as unproblematic and not considered likely to lead to regular use or tobacco smoking. Trying an e-cigarette was driven by various factors, including social gains from sharing with peers, fun, and appeal of flavours:

Yeah and you can get them like scented and stuff like that and so it entices young people. (W1(2)F)

I just liked the different flavours. Cos my friend had jam donut, another friend had gummy bear flavour and Heisenberg which is a minty flavour which is quite nice. (S1(1)M)

Regular e-cigarette use was judged negatively however. When asked to consider who regular users were among their school cohort, views were mixed. Some felt that regular users came from the same groups as smokers, characterised as ‘outsiders’ or disengaged pupils. Others described smokers and e-cigarette users as distinct populations, including e-cigarette users being non-smoking pupils performing tricks. Tricks were of significant interest and had been viewed by almost all, predominantly on social media, with imitation motivating use, particularly among boys. Although experimentation with e-cigarettes was very evident at this point, it was also a discussion point in many groups that this behaviour was a trend that had peaked and was likely to decrease among their cohort going forward:

Like the fidget spinners, and then I think as soon as fidget spinners die it's going to be the same kind of, “Oh why are you still using them?” kind of approach, the same as vaping. (E1(1)M)

To explore this purported peak, the question of prevalence was discussed in repeat interviews in 2018, several months after our quantitative time series data. This appeared to confirm initial indications of e-cigarettes as a ‘fad’ that had peaked and become of less interest:

…you'll see things of it occasionally but I don't see anyone doing it just to show off now, any more. (W1(2)M)

E-cigarettes were now described as being more prevalent in young adults who were using them to quit smoking, or younger pupils who were experimenting and still attracted by the flavours and features that had drawn interviewees to try them previously. In 2018, approval of any e-cigarette use was more variable than in 2017 (when acceptance was widespread), with increased likelihood of peer censure:

They just go – (mocking tone) I've got a vape, I'm sick, look at me! (E4(3)F)

I know some of the kids in our year they're like, oh they just made jokes about it, oh you're vaping, and think it's a bit chavvy, and some, they think it's cool. (W4(1)F)

When initially discussing reasons for use in 2017, very few cited quitting smoking as a driver for young people's e-cigarette use. Instead, this was confined to reasons proposed for adult use, reflecting the perceived rarity of regular smoking at this age. However, in 2018 follow-up interviews, this was cited more frequently as a reason why young people may use e-cigarettes; with a perceived growth in smoking associated with maturation. Regular e-cigarette use for this purpose was largely approved, in contrast with regular use in non-smokers which was not:

I think that if it's used to actually try and give up smoking then I see a huge advantage to it, but obviously if you're videoing yourself doing tricks and stuff that's not trying to give it up (smoking), is it? (W3(2)F)

#### Risk perception

In 2017, increased e-cigarette visibility was widely stated as being instrumental in increasing acceptability and reducing risk perceptions. However, while a vast majority identified e-cigarettes as less harmful than smoking, they were not considered harm-free. When asked to discuss the types of risk associated with e-cigarette use, pupils frequently cited: mechanical risks (e.g. malfunctioning devices); potentially dangerous ingredients in liquids; and, most commonly, as yet unidentified harms:

Because they know what's in the cigarette, like in tobacco cigarettes but they don't know what's fully in the e-cigarettes or the vapes. (W2(3)M)

Quantitative data from Wales, post-implementation (Table 5), indicated that approximately half of young people perceived e-cigarettes as less harmful than tobacco. While few perceived them as worse, around a third perceived them to be equally as harmful. Notably, ever-smokers were more likely to perceive e-cigarettes as more harmful than tobacco.

**Table 5:** Health risk perceptions for tobacco and e-cigarette use, and content of last e-cigarette used, among 13 and 15 year olds in Wales in 2017

	Perceived harms of smoking and vaping				Nicotine content of last e-cigarette used (ever users only)				
	Smoking worse (%)	Vaping worse	Equally harmful	Don't know	Nicotine	Flavour / water vapour only	Cannabis or cannabis oil	Something else	Don't know
All	6,132 (47.8)	388 (3.0)	4,529 (35.3)	1,775 (13.8)	2,659 (29.9)	4,681 (52.7)	247 (2.8)	80 (0.9)	1,220 (13.7)
Boys	3,442 (54.8)	181 (2.9)	1,831 (29.2)	825 (13.1)	1,215 (26.5)	2,644 (57.7)	151 (3.3)	50 (1.1)	521 (11.4)
Girls	2,579 (41.0)	192 (3.1)	2,627 (41.8)	891 (14.2)	1,371 (33.5)	1,949 (47.7)	70 (1.7)	26 (0.6)	672 (16.4)
Year 9	3,376 (45.0)	213 (2.8)	2,783 (37.1)	1,123 (15.0)	937 (25.5)	2,057 (56.0)	86 (2.3)	37 (1.0)	555 (15.1)
Year 11	2,756 (51.7)	175 (3.3)	1,746 (32.8)	652 (12.2)	1,722 (33.0)	2,624 (50.3)	161 (3.1)	43 (0.8)	665 (12.8)
Never-smoker	4,800 (46.4)	216 (2.1)	3,902 (36.7)	1,429 (13.8)	772 (17.0)	2,959 (65.2)	50 (1.1)	34 (0.8)	725 (16.0)
Ever- smoker	1,204 (54.8)	150 (6.8)	565 (25.7)	277 (12.6)	1,769 (44.4)	1,548 (38.9)	183 (4.6)	43 (1.1)	442 (11.1)

Note: Sample sizes differ for perceived harm (n=12,824) and content of last e-cigarette (n=8,887). Perceived harm was asked of a random subsample of pupils, whereas content of last e-cigarette was asked of a sample of ever users only. Both samples include gender non-response (n=256, 2.0%; n=218, 2.5%)

In both 2017 and 2018 qualitative interviews, mechanical risks and unknown harms in relation to e-cigarettes were commonly referenced during group discussions. In 2017, addiction to nicotine was also mentioned in around two thirds of groups as a risk of e-cigarette use. However, in 2018 a reduced proportion of young people mentioned addiction to nicotine compared to the previous year. Many young people who had used an e-cigarette said they were unsure if it had contained nicotine or not, with use driven more by flavour than nicotine. However, quantitative data from Wales, post-TPD implementation (Table 5), indicated that most reported awareness of the content of the last device they used. In a minority of cases were these reported to contain nicotine. This was highly patterned by smoking status, with a sixth of never smokers reporting use of devices containing nicotine compared to almost half of ever-smokers.

Risk was further explored through perceived family and peer reactions to e-cigarette or tobacco smoking adoption. In 2017, most participants reported that their parents would strongly disapprove of them using either e-cigarettes or tobacco, with almost all suggesting a worse reaction to tobacco. Some interpreted this as parental fear of moving on to other substances such as cannabis, while others suggested it being due to harms associated with smoking being better known than for vaping:

Because if you're smoking (vape) they'll probably think you're smoking everything else. (E2(2)F)

INT: If it was tobacco would it be a similar reaction or worse (to vaping)?

R: Much worse…My parents would not be happy. Because they know the damage it does to your body … My mum's a nurse and she knows all about it, and it wouldn't be very approved of in my family, because of the damage it causes to your lungs, really. (W3(2)F)

Perceived levels of parental disapproval for both behaviours remained high in 2018 and most pupils continued to state that disapproval of smoking would be higher than that of e-cigarettes. Quantitative data from Wales in 2017 concurred with this perception (Table 6).

**Table 6:** Perceptions of parental attitudes to regulation of smoking and e-cigarette use among 13 and 15 year olds in Wales in 2017

		Try to stop me (%)	Try persuading me to stop	Do nothing	Encourage me to vape
E-cigarettes (non-users)	All *(n=11,197; 100.0%)*	8,365 (74.7)	2,414 (21.6)	345 (3.1)	73 (0.6)
	Boys *(n=5,427; 48.5%)*	3,969 (73.1)	1,216 (22.4)	195 (3.6)	47 (0.9)
	Girls *(n=5,597; 50.0%)*	4,274 (76.4)	1,163 (20.8)	137 (2.4)	23 (0.4)
	Year 9 *(n=6,622; 59.1%)*	5,234 (79.0)	1,224 (18.5)	134 (2.0)	30 (0.4)
	Year 11 *(n=4,575; 40.9%)*	3,131 (68.4)	1,190 (26.0)	211 (4.6)	43 (0.9)
Smoking (non-smokers)	All *(n=11,442; 100.0%)*	9,774 (85.4)	1,495 (13.1)	133 (1.2)	40 (0.4)
	Boys *(n=5,661; 49.5%)*	4,827 (85.3)	739 (13.1)	68 (1.2)	27 (0.5)
	Girls *(n=5,592; 48.9%)*	4,796 (85.8)	733 (13.1)	57 (1.0)	6 (0.1)
	Year 9 *(n=6,905; 60.4%)*	6,018 (87.2)	799 (11.6)	63 (0.9)	25 (0.4)
	Year 11 *(n=4,537; 39.7%)*	3,756 (82.8)	696 (15.3)	70 (1.5)	15 (0.3)

Note: Sample includes gender non-response (e-cigarettes: n=173, 1.5%; smoking: n=189, 1.6%)

Perceived peer reactions were more varied: 2017 interviews described some expectation of negative peer reaction to both smoking and e-cigarette use but at lower levels than that expected from parents. A majority also suggested that peers would be more likely to censure any smoking than any use of e-cigarettes; however, this changed when the same pupils were re-interviewed in 2018, with a general decrease in disapproval of ‘social’ smoking (but retention of disapproval of regular smoking):

I think it is more socially accepted like, now, like in our year to smoke than it was last year. Like you see a lot more people smoking than before especially at parties. (E1(1)M)

#### Availability of e-cigarettes

In 2017, most pupils agreed that they would find it easier to obtain e-cigarettes than tobacco cigarettes, with only a small number suggesting that both were equally obtainable:

But, like, e-cigarettes, it seems to be that you can get hold of one quite easily. But cigarettes – you have to like, know somebody who's old enough to go buy you some. (S1(2)F)

For most interviewees who had tried an e-cigarette, this had been through an informal supply route (e.g. a friend at a social event), although some reported awareness of someone at school who had bulk bought online with the intention to sell them on:

I was also one of the people who were making profit from them, people would ask me could you order one for me, and I was like okay well I'll order them. I'd order ten at once. (E1(1)M)

A very small number stated that e-cigarettes were available through retailers. Qualitative data indicated that when purchased face-to-face, this was through shops known locally to be willing to sell illicit products rather than via specialist vape shops:

Like I've seen down in XXXX market, they sell to someone who was 9. (W2(3)M)

Quantitative data from Wales in 2017 concurred with the view that most young people who used e-cigarettes obtained them via means other than purchasing from a retailer: 31.7% of young people reported obtaining e-cigarettes from peers, for example, compared to 15.2% from shops (Table 7).

**Table 7:** Modes of obtaining e-cigarettes among 13 and 15 year olds in Wales in 2017

	Buy from shop (%)	Internet	From adults	From peers	Take	From siblings	Other
All *(n=3,313; 100.0%)*	505 (15.2)	227 (6.9)	553 (16.7)	1,049 (31.7)	285 (8.6)	129 (3.9)	654 (19.7)
Boys *(n=1,840; 55.5%)*	367 (20.0)	164 (8.9)	279 (15.2)	550 (29.9)	150 (8.2)	65 (3.5)	378 (20.5)
Girls *(n=1,342; 40.5%)*	111 (8.3)	53 (4.0)	254 (18.9)	469 (35.0)	112 (8.4)	53 (4.0)	249 (18.6)
Year 9 *(n=1,413; 42.7%)*	138 (9.8)	59 (4.2)	196 (13.9)	474 (33.6)	136 (9.6)	54 (3.8)	293 (20.7)
Year 11 *(n=1,900; 57.4%)*	367 (19.3)	168 (8.8)	357 (18.8)	575 (30.3)	149 (7.8)	75 (4.0)	361 (19.0)
Never smoker *(n=887; 28.4%)*	77 (8.7)	44 (5.0)	93 (10.5)	287 (32.4)	52 (5.9)	23 (2.6)	150 (16.9)
Ever smoker *(n=2,233; 71.6%)*	405 (18.1)	173 (7.8)	436 (19.5)	731 (32.7)	213 (9.5)	101 (4.6)	467 (20.9)

Note: multiple responses allowed. Figures therefore reflect numbers of pupils that acquired e-cigarettes via each method (and as a percentage of the total sample). Sample includes gender non-response (n=131; 4.0%)

Discussions a year later suggested that the landscape had become quite different. Many pupils still felt that they could obtain e-cigarettes if desired, mostly through older peers or online purchase, but it was seen as more challenging to buy through school supply chains. This was associated with a perception of decreased use, which meant less people now selling them than before:

INT: do you think it's got easier or harder to get hold of vapes?

R: I think it's a bit harder because you don't really see that many people do it anymore.

INT: …people selling them, would that go on in school or after school?

R2: I don't think it's much in school. I know some people used to do it (sell vapes), but I don't know anyone now who does it.

(W1(1)M)

Tobacco was viewed as being as easy to obtain as e-cigarettes and easier than the previous year. This was attributed to factors such as increased age, which meant greater likelihood either of having older peers or appearing old enough to self-purchase in shops, but also to increased smoking prevalence in the year group; and hence a greater number of people to obtain tobacco from if desired. A substantial number were aware of someone at school who sold individual cigarettes, making them an affordable option:

I know in school obviously a lot of people do it in the corner, but some people sell them individually for 50 pence or maybe a £1 or something, and people do pay that money for them individually. (W3(2)F)

#### Exposure to warning information and e-cigarette marketing

TPD introduced a mandated warning stating ‘this product contains nicotine, which is a highly addictive substance’. In 2017, recall of this warning among interviewees was low. This may have been due to non-compliant products still being available on the market at this time, but it could also have been associated with informal supply routes, where packaging was rarely seen. In 2018, recognition of the nicotine message slightly increased, but exposure to packaging remained limited:

INT: Have you Inconsistent indentation seen any warnings on vape packets?

R: *No. Can you even get vape packets?* (E4(1)F)

This further supports the notion that experimental users may be unaware of the potential nicotine content of liquids, relying on the device owner to supply this information.

When asked to discuss the current warning, a large majority suggested it would likely be ineffective at discouraging e-cigarette use among their age group. Pupils commonly assessed the warning in relation to more graphic and more well-known visuals present on tobacco packaging, deeming e-cigarette warnings mild by comparison:

Like if I was vaping and like I was like “oh it's fine because look at how bad cigarettes were, like I'm fine”, because there's no health warnings on any of the (e-cigarette) packets. (S2(2)F)

Brand awareness was very low; likely owing to informal supply chains, where product packaging was rarely seen, coupled with low levels of reported exposure to e-cigarette advertising. The most commonly reported avenue for seeing adverts in 2017 was through high street vape shops, followed by online sources (usually streamed videos of e-cigarette users performing tricks). Exposure to advertising in public spaces such as billboards was also seen as relatively common. Across the 2018 interviews, there was decreased reporting of seeing any e-cigarette advertising.

Quantitative data from Wales in 2017 indicated that most young people recalled some form of e-cigarette marketing exposure in the previous month (66.8%), with around 40% exposed to marketing online or via point of sale displays (Table 8). See Supplementary Tables 14-17 for process indicators by ethnicity and SES.

**Table 8:** Prevalence and locations of exposure to e-cigarette marketing among 13 and 15 year olds in Wales in 2017

	Bus shelter (%)	Side of bus	Billboard	Supermarket, petrol station, newsagent vape shop	Internet	Phone box	Other	No exposure
All *(n=13,503; 100.0%)*	2,793 (20.7)	1,492 (11.1)	1,997 (14.8)	5,604 (41.5)	5,261 (39.0)	1,067 (7.9)	2,509 (18.6)	4,494 (33.2)
Boys *(n=6,655; 49.3%)*	1,425 (21.4*)*	825 (12.4)	1,026 (15.4)	2,585 (38.8)	2,555 (38.4)	539 (8.1)	1,325 (19.9)	2,276 (34.2)
Girls *(n=6,542; 48.5%)*	1,276 (19.5)	603 (9.2)	896 (13.7)	2,905 (44.4)	2,592 (39.6)	471 (7.2)	1,105 (16.9)	2,134 (32.6)
Year 9 *(n=7,912; 58.6%)*	1,631 (20.6)	879 (11.1)	1,140 (14.4)	3,238 (40.9)	2,896 (36.6)	614 (7.8)	1,665 (21.0)	2,692 (34.0)
Year 11 *(n=5,591; 41.4%)*	1,162 (20.8)	613 (11.0)	857 (15.3)	2,366 (42.3)	2,365 (42.3)	453 (8.1)	844 (15.1)	1,802 (32.2)
Never smoker *(n=10,539; 82.3%)*	2,154 (20.4)	1,101 (10.5)	1,531 (14.5)	4,477 (42.5)	4,120 (39.1)	775 (7.4)	1,993 (18.9)	3,748 (35.6)
Ever smoker *(n=2,268; 17.7%)*	557 (24.6)	350 (15.4)	411 (18.1)	1,020 (45.0)	1,026 (45.2)	261 (11.5)	446 (19.7)	641 (28.3)

Note: multiple responses allowed. Figures therefore reflect numbers and percentages of the total sample of pupils exposed to e-cigarette marketing via each mechanism. Sample includes gender non-response (n=306; 2.3%)

## Discussion

Internationally, regulatory frameworks for e-cigarettes continue to diverge, with a range of recent policy actions motivated by limiting young people's exposure to e-cigarettes. India for example recently passed a ban on sales of e-cigarettes, due to concerns regarding a perceived youth epidemic (BBC News, 2019). Moves such as flavour bans have been debated widely in the US; advocates argue that this would reduce appeal to children (Doward & Fraser, 2019), while critics argue it would make adults more likely to choose tobacco cigarettes (Buckell, Marti, & Sindelar, 2019). This study provides some evidence that in UK nations, growth in young people's experimentation with e-cigarettes has begun to slow, following introduction of EU regulations which limited marketing, nicotine strength and mandated product labelling.

Before interpreting the contribution of this study, a number of strengths and limitations merit consideration. Data are based on self-reports, prone to social desirability biases which may change over time as behaviours become more or less normalised. Use of separate surveys in England and Wales meant items could not be harmonised, limiting between country comparisons. Survey timings were not synchronised, and post-implementation data from England were collected only a few months after the policy implementation date, while pre-implementation data were collected 18 months prior. Hence, the proportion of change occurring in the 18 months prior to regulation, rather than beyond it, cannot be estimated. Our initial intention was to include data from the Scottish Schools’ Adolescents Lifestyle and Substance Use Survey, but postponement of the 2017 survey meant no post-implementation data were available. Only in Wales was it possible to undertake a more robust interrupted time-series analysis which adjusted for trend prior to implementation.

Comparisons within and between countries may also be impacted by response rate trajectories. While pupil responses to school based surveys are typically high, school level response rates to social surveys have declined in recent years. The SDDU survey experienced a declining school response rate from 40% in 2014 to 26% in 2016. In Wales, response rates to HBSC declined to 2013, such that only 46% of schools approached agreed to participate. However, in 2013, schools were offered the option to join a network who would participate biennially and receive bespoke data on wellbeing of pupils in their school, with network events to support schools in using data to plan health improvement action (Murphy, et al., 2018). Most participated again in 2015, with additional schools recruited to ensure representation of local authorities from whom no schools were recruited for the 2013 survey. By 2017, the school-level response rate was increased to 91% through this more engaged model. Hence, later surveys in Wales are likely subject to lower response bias than earlier surveys, while the inverse was true in England. Response bias may lead to underestimation of substance use outcomes, and if earlier values underestimate prevalence while post-implementation estimates were more accurate, subsequent growth may have been exaggerated. Reassuringly, in Wales, post-hoc analysis harmonising the sample by using only data from schools providing data for all surveys showed a similar pattern to the main analysis.

To enhance causal inference, energy drink use was selected for falsifiability checks as an emerging public health issue which, like e-cigarettes, was not following the secular decline observed for most substances, but was unlikely to be impacted by TPD. However, energy drink use has also become a contentious public health issue, with products impacted by the soft drinks industry levy (BBC, 2016), and UK governments considering age of sale restrictions (Department for Health and Social Care, 2018). Hence, change in use may have occurred around the same time as TPD, albeit for differing reasons. Timing of obtaining funding meant that it was not possible to collect qualitative data from young people prior to regulations, with these conducted longitudinally from the transitional period through to longer term implementation. At the behest of our ethics committee, we initially used opt-in parental consent for interviews with young people; a process which often leads to under-representation of higher risk groups of young people, whose perspectives on vaping may have differed from the sample recruited (Liu, Cox, Washburn, Croff, & Crethar, 2017). For additional schools recruited in 2018, a more standard opt-out process was used, with few striking differences in themes identified.

Notwithstanding these limitations, the study is strengthened by use of large representative samples of young people in England and Wales, combined with qualitative insights from purposive samples of young people to aid interpretation of trend data, and offers important insights into use patterns and mechanisms. While first measured in 2013 in Wales and 2014 in England, given that use of e-cigarettes among UK adults began to emerge from 2011 (West, et al., 2018), young people's experimentation was likely negligible until that point. Hence, prevalence of ever use in Wales and England grew from close to zero in 2011 to approximately 1 in 4 in England in 2014 and Wales by 2015, increasing more marginally in the following post-implementation surveys. In Wales, segmented regression models, assuming uninterrupted growth until TPD implementation provided some evidence of plateauing post-implementation. Where holding smoking status constant across the time series, change in trend for ever e-cigarette use became larger, suggesting that impact on use of e-cigarettes may have been suppressed in a priori models by underlying changes in ever smoking across the time series. However, we cannot rule out collider bias in that adjustment by smoking may have induced a spurious association between TPD and e-cigarette trend change (Elwert & Winship, 2014). Simpler before and after analysis of weekly e-cigarette use from 2015 to 2017 in Wales (measured for the first time in 2015) indicated only marginal further increase. Although data from England indicated that weekly use continued to increase between 2014 and 2016, newly published SDDU data from 2018, unavailable for our analysis at the time of writing but which will be included in our longer term analysis, reported no growth in experimental or regular e-cigarette use in England from 2016 to 2018 (National Statistics, 2019). Similarly, recent estimates from SALSUS surveys showed marginal growth in experimental use only in Scotland from 2015 to 2018 (NHS Digital, 2019).

Overall, evidence of change in trajectory for e-cigarette experimentation remains tentative and sensitive to model assumptions. Changes in trend for energy drink use were similar to those for e-cigarettes, and if this mirroring of trend by an unrelated outcome continues in longer term analysis, this somewhat undermines our confidence that change in trend is causally related to TPD. However, our data, combined with estimates published elsewhere as described above, appear to provide an early signal that young people's use of e-cigarettes reached its peak around the time of TPD implementation.

Process data allowed us to unpack mechanisms associated with trends in young people's use of e-cigarettes through the transitional phase for TPD regulations and beyond. Continued growth in experimental vaping between 2015 and 2017, coupled with more limited growth of regular use, is consistent with qualitative data suggesting widespread social approval of experimental vaping in social situations such as parties, but simultaneous disapproval of regular use (Katz, Erkinnen, Lindgren, & Hatsukami, 2019). Our confidence in the causal nature of any disruption to the rise in experimentation with e-cigarettes arising from the TPD regulation was challenged by a strong emerging theme which centred around vaping as a fad which was beginning to have run its course, like adult vaping, reaching a plateau (West, et al., 2018).

In relation to risk perceptions, targeted by TPD via product labelling, qualitative data suggested young people viewed e-cigarettes as harmful, though less so than tobacco (Berg, et al., 2015; Weishaar, et al., 2016). Quantitative survey data shortly after regulations were introduced in Wales indicated wide divergence of opinion with half of young people perceiving tobacco to be more harmful than e-cigarettes. Only a small proportion believed e-cigarettes more harmful than tobacco, although this was substantially more common among ever-smokers. Notably, 1 in 3 stated that both were equally harmful. While the primary mechanism within TPD for changing risk perceptions was product labelling, qualitative data suggested that most young people could not recall seeing the messages; many described never having seen e-cigarettes in their packaging, with e-cigarettes primarily used socially at parties and passed between peers, rather than young people each buying their own devices (Katz, et al., 2019). Quantitative data reinforced this finding that young people who used e-cigarettes tended not to purchase them via retail and were more likely to come into contact with them via their networks.

In qualitative interviews, young people demonstrated little recall of product or brand advertising. Notably, while product regulation came into force gradually, advertising restrictions were already in force prior to initial qualitative interviews. However, young people reported continuing to interact with online material primarily streaming videos of e-cigarette users doing tricks which were often imitated in social gatherings. Point of sale displays and the internet, described prior to TPD as key sources of influence on young people's experimentation with e-cigarettes (Best, et al., 2016; Singh, et al., 2016), continue to be widespread sources of exposure to e-cigarette promotions after TPD, with around two-fifths of young people reporting exposure via both these avenues.

Concerns regarding young people's use of e-cigarettes have centred largely on assumptions that this exposes young people to nicotine, which may lead to nicotine addiction and risk of transitioning into tobacco use (Chapman, et al., 2018; Conner, et al., 2018). However, surveys have not asked young people to differentiate between products which do, or do not, contain nicotine. In qualitative interviews, nicotine was described infrequently by young people as a motivator for use of e-cigarettes, although the phenomenon of young people accessing e-cigarettes without having seen packaging raised significant concerns regarding whether young people were unknowingly using nicotine. In Wales in 2017, most young people who had used e-cigarettes reported that they did not contain nicotine. Nicotine-free devices were more popular with never-smokers, and vice versa for ever smokers. Hence, few never-smoking young people reported exposure to nicotine via e-cigarettes, and nicotine strength regulations may have had minimal impact on use of products which were on the market at the time of regulation. However, a potentially important impact of regulation has been blocking entry to the market of high strength devices which have gained traction among young people in US markets (Hammond, et al., 2019; Kavuluru, et al., 2019), as well as limiting the capacity for these devices to be positioned as lifestyle devices via regulation of marketing.

### Conclusions and directions for future research

The study provides tentative early indications that previously rapid growth in young people's experimentation with e-cigarettes may now be slowing. Young people's perceptions offered a range of causal explanations for the slowed increase in e-cigarette use, including it being a passing fad which had begun to run its course. The social dynamics and supply mechanisms described by young people meant that exposure to aspects of TPD such as product labelling was minimal, while substantial proportions of young people continued to be exposed to a range of forms of e-cigarette marketing after TPD. Longer term data are needed to capture whether experimentation has now plateaued, gone into decline, or will rise once again as newer products enter the market. There is a notable contrast in our data and those from North America, where growth in e-cigarette use has continued to accelerate, as high nicotine strength products have gained traction. It is likely that regulations have played a role in slowing entry of these products to EU markets such as the UK and limiting their marketing. These analyses form part of a larger study which will include longer term analyses of quantitative trends, and qualitative analyses of data from retailers, trading standards officers and policy stakeholders. This will provide further understandings of processes of change initiated by regulations in context. This study did not focus on tobacco use, other than through association with patterns of e-cigarette use over time; post TPD changes in smoking rates will also be examined in subsequent analysis drawing on the longer time series of smoking data across countries. Prevention of young people's smoking uptake and e-cigarette use remain important foci for public health intervention.

## Declarations of Interests

LB declares a secondment post with Cancer Research UK. All other authors have no conflicts of interest to declare.

## Appendix Supplementary materials

Image, application 1
