# Supplementary material for: Young people's use of e-cigarettes in Wales, England and Scotland before and after introduction of EU Tobacco Products Directive regulations: a mixed-method natural experimental evaluation
Source: Int J Drug Policy. 2020 Nov;85:102795. doi: 10.1016/j.drugpo.2020.102795 (PMC7773804; doi:10.1016/j.drugpo.2020.102795)
Supplement: Supplementary file 1 [file mmc1.docx]

**Supplementary material**

Figure 1: Predicted probabilities for ever use of e-cigarettes by age and sex for period November 2013 to December 2017 from models including a quadratic time trend (intervention point: May 2016)

Table 1: Prevalence and CIs (95%) of ever e-cigarette use among pupils in Wales between November 2013 and December 2017, overall and by sex and school year

|  |  |  | 95% CI | |  | 95% CI | |  | 95% CI | |  | 95% CI | |  | 95% CI | |
| --- | --- | --- | --- | --- | --- | --- | --- | --- | --- | --- | --- | --- | --- | --- | --- | --- |
| Month-Year | Time | **Total**  **(%)** | Low | High | **Boys**  **(%)** | Low | High | **Girls**  **(%)** | Low | High | **Year 9 (%)** | Low | High | **Year 11**  **(%)** | Low | High |
| 11/2013 | 0 | 17.6 | 15.5 | 19.8 | 16.6 | 13.8 | 19.7 | 18.6 | 15.7 | 21.9 | 11.1 | 8.8 | 13.7 | 24.8 | 21.4 | 28.4 |
| 12/2013 | 1 | 16.5 | 14.3 | 19.0 | 18.7 | 15.4 | 22.4 | 14.0 | 11.0 | 17.5 | 11.5 | 8.9 | 14.5 | 22.5 | 18.7 | 26.6 |
| 01/2014 | 2 | 15.8 | 13.3 | 18.6 | 17.3 | 13.7 | 21.5 | 14.1 | 10.7 | 18.1 | 12.7 | 9.7 | 16.2 | 19.8 | 15.6 | 24.5 |
| 02/2014 | 3 | 13.3 | 10.2 | 16.8 | 12.6 | 8.7 | 17.5 | 13.8 | 9.4 | 19.3 | 10.3 | 6.9 | 14.6 | 17.5 | 12.3 | 23.8 |
| 03/2014 | 4 | 22.7 | 7.8 | 45.4 | 38.5 | 13.9 | 68.4 | *** | *** | *** | - | - | - | 22.7 | 7.8 | 45.4 |
| 09/2015 | 22 | 27.8 | 25.5 | 30.2 | 29.3 | 25.7 | 33.1 | 26.7 | 23.7 | 29.8 | 22.3 | 19.5 | 25.4 | 34.3 | 30.7 | 38.1 |
| 10/2015 | 23 | 28.8 | 27.4 | 30.3 | 28.7 | 26.7 | 30.8 | 28.9 | 27.0 | 30.9 | 21.3 | 19.7 | 23.0 | 41.1 | 38.7 | 43.7 |
| 11/2015 | 24 | 31.6 | 30.3 | 32.8 | 31.9 | 30.1 | 33.8 | 31.2 | 29.5 | 33.0 | 22.5 | 20.9 | 24.1 | 40.4 | 38.6 | 42.3 |
| 09/2017 | 46 | 32.5 | 30.8 | 34.3 | 35.0 | 32.5 | 37.7 | 30.3 | 28.0 | 32.7 | 20.6 | 18.4 | 23.0 | 41.7 | 39.2 | 44.1 |
| 10/2017 | 47 | 35.8 | 34.8 | 36.8 | 38.4 | 37.0 | 39.8 | 33.2 | 31.9 | 34.6 | 27.4 | 26.3 | 28.6 | 47.5 | 45.9 | 49.1 |
| 11/2017 | 48 | 34.8 | 33.9 | 35.6 | 38.5 | 37.2 | 39.8 | 30.9 | 29.7 | 32.2 | 27.8 | 26.8 | 28.9 | 45.4 | 43.9 | 46.9 |
| 12/2017 | 49 | 34.0 | 33.2 | 34.8 | 35.1 | 34.0 | 36.3 | 32.9 | 31.8 | 34.0 | 24.2 | 23.2 | 25.2 | 45.3 | 44.1 | 46.6 |

***Prevalence rate and CIs not given for this time point due to small number of observations (n=9)**.** Inclusion of this time point in regression analyses did not adversely impact modelled estimates.

Table 2: Odds ratios (with 95% CIs) for ever use of e-cigarettes among pupils in Wales between 2013 and 2017 by ethnicity and socioeconomic status (segmented regression analyses)

|  |  | Ever used e-cigarettes | | | |
| --- | --- | --- | --- | --- | --- |
|  |  | Linear models | *P* | Quadratic models | *P* |
| White  (n=45,727) | Time (month-year) | 1.04 [1.03, 1.05] | <0.001 | 1.01 [0.89, 1.15] | 0.880 |
|  | Time^2^ | - | - | 1.00 [1.00, 1.01] | 0.677 |
|  | Level | 1.19 [0.45, 3.15] | 0.726 | 1.37 [0.43, 4.31] | 0.593 |
|  | Post-slope | 0.95 [0.90, 1.01] | 0.089 | 0.88 [0.60, 1.28] | 0.506 |
| BME  (n=4,528) | Time | 1.03 [1.01, 1.05] | 0.001 | 0.91 [0.56, 1.48] | 0.702 |
|  | Time^2^ | - | - | 1.01 [0.99, 1.02] | 0.602 |
|  | Level | 0.56 [0.04, 7.35] | 0.662 | 1.06 [0.03, 40.90] | 0.975 |
|  | Post-slope | 1.00 [0.86, 1.15] | 0.963 | 0.69 [0.17, 2.76] | 0.600 |
| High SES  (n=25,002) | Time | 1.04 [1.03, 1.05] | <0.001 | 1.05 [0.89, 1.23] | 0.554 |
|  | Time^2^ | - | - | 1.00 [0.99, 1.01] | 0.887 |
|  | Level | 1.76 [0.62, 4.97] | 0.285 | 1.66 [0.47, 5.91] | 0.432 |
|  | Post-slope | 0.94 [0.88, 1.00] | 0.037 | 0.97 [0.61, 1.53] | 0.897 |
| Low SES  (n=25,167) | Time | 1.04 [1.03, 1.05] | <0.001 | 0.99 [0.86, 1.14] | 0.900 |
|  | Time^2^ | - | - | 1.00 [1.00, 1.01] | 0.525 |
|  | Level | 0.73 [0.23, 2.28] | 0.592 | 0.92 [0.25, 3.43] | 0.901 |
|  | Post-slope | 0.98 [0.92, 1.04] | 0.500 | 0.86 [0.57, 1.29] | 0.461 |

Note: models adjusted for sex and school year

Table 3: Odds ratios (with 95% CIs) for ever use of e-cigarettes among pupils in Wales between 2013 and 2017 with smoking status (ever smoked = 1) as a modelled covariate (segmented regression analyses)

|  |  | Ever used e-cigarettes | | | |
| --- | --- | --- | --- | --- | --- |
|  |  | Linear models | *P* | Quadratic models | *P* |
| All  (n=49,449) | Time (month-year) | 1.07 [1.06, 1.08] | <0.001 | 1.08 [0.94, 1.25] | 0.280 |
|  | Time^2^ | - | - | 1.00 [0.99, 1.01] | 0.832 |
|  | Level | 1.09 [0.42, 2.82] | 0.867 | 1.00 [0.30, 3.34] | 0.994 |
|  | Post-slope | 0.93 [0.88, 0.98] | 0.005 | 0.97 [0.64, 0.73] | 0.879 |
|  | Smoking status | 23.31 [21.36, 25.43] | <0.001 | 23.32 [21.38, 25.43] | <0.001 |
| Boys  (n=24,085) | Time (month-year) | 1.06 [1.04, 1.07] | <0.001 | 1.09 [0.93, 1.28] | 0.272 |
|  | Time^2^ | - | - | 1.00 [0.99, 1.00] | 0.673 |
|  | Level | 1.68 [0.63, 1.49] | 0.304 | 1.41 [0.40, 4.94] | 0.587 |
|  | Post-slope | 0.92 [0.87, 0.97] | 0.003 | 1.01 [0.65, 1.59] | 0.955 |
|  | Smoking status | 21.04 [18.78, 23.57] | <0.001 | 21.06 [18.80, 23.59] | <0.001 |
| Girls  (n=25,364) | Time (month-year) | 1.07 [1.06, 1.09] | <0.001 | 1.06 [0.88, 1.29] | 0.515 |
|  | Time^2^ | - | - | 1.00 [0.99, 1.01] | 0.923 |
|  | Level | 0.68 [0.18, 2.55] | 0.569 | 0.71 [0.13, 3.79] | 0.692 |
|  | Post-slope | 0.93 [0.87, 1.01] | 0.070 | 0.91 [0.52, 1.59] | 0.739 |
|  | Smoking status | 25.10 [22.48, 28.03] | <0.001 | 25.10 [22.49, 28.02] | <0.001 |
| Year 9  (n=27,543) | Time (month-year) | 1.06 [1.05, 1.08] | <0.001 | 1.13 [0.95, 1.34] | 0.182 |
|  | Time^2^ | - | - | 1.00 [0.99, 1.00] | 0.518 |
|  | Level | 1.68 [0.43, 6.61] | 0.459 | 1.25 [0.25, 6.16] | 0.784 |
|  | Post-slope | 0.90 [0.84, 0.97] | 0.009 | 1.07 [0.64, 1.80] | 0.797 |
|  | Smoking status | 35.81 [31.57, 40.61] | <0.001 | 35.84 [31.58, 40.66] | <0.001 |
| Year 11  (n=21,906) | Time (month-year) | 1.07 [1.05, 1.08] | <0.001 | 1.03 [0.87, 1.22] | 0.739 |
|  | Time^2^ | - | - | 1.00 [0.99, 1.01] | 0.680 |
|  | Level | 0.77 [0.23, 2.55] | 0.664 | 0.91 [0.21, 4.16] | 0.907 |
|  | Post-slope | 0.95 [0.89, 1.01] | 0.104 | 0.86 [0.52, 1.40] | 0.531 |
|  | Smoking status | 17.95 [16.15, 19.97] | <0.001 | 17.94 [16.14, 19.95] | <0.001 |

Note: where appropriate, models adjusted for sex and school year

Table 4: Odds ratios (with 95% CIs) for ever use of e-cigarettes among pupils in Wales between 2013 and 2017, overall and by sex, school year and smoking status – quadratic models (segmented regression analyses)

|  |  |  | *P* |
| --- | --- | --- | --- |
| All  (n=51,056) | Time (month-year) | 1.00 [0.87, 1.15] | 0.994 |
|  | Time^2^ | 1.00 [1.00, 1.01] | 0.589 |
|  | Level | 1.32 [0.41, 4.21] | 0.640 |
|  | Post-slope | 0.86 [0.58, 1.27] | 0.451 |
| Boys  (n=24,993) | Time | 1.03 [0.90, 1.19] | 0.654 |
|  | Time^2^ | 1.00 [0.99, 1.01] | 0.960 |
|  | Level | 1.91 [0.57, 6.37] | 0.292 |
|  | Post-slope | 0.93 [0.62, 1.38] | 0.707 |
| Girls  (n=26,063) | Time | 0.97 [0.81, 1.15] | 0.706 |
|  | Time^2^ | 1.00 [1.00, 1.01] | 0.425 |
|  | Level | 0.92 [0.21, 3.97] | 0.916 |
|  | Post-slope | 0.80 [0.48, 1.33] | 0.381 |
| Year 9 (n=28,471) | Time | 1.07 [0.90, 1.26] | 0.461 |
|  | Time^2^ | 1.00 [0.99, 1.01] | 0.745 |
|  | Level | 1.29 [0.27, 6.10] | 0.750 |
|  | Post-slope | 1.02 [0.62, 1.68] | 0.926 |
| Year 11  (n=22,585) | Time | 0.95 [0.81, 1.11] | 0.513 |
|  | Time^2^ | 1.00 [1.00, 1.01] | 0.255 |
|  | Level | 1.34 [0.33, 5.52] | 0.684 |
|  | Post-slope | 0.75 [0.48, 1.17] | 0.206 |
| Never smoker (n=40,703) | Time | 1.06 [0.90, 1.24] | 0.459 |
|  | Time^2^ | 1.00 [0.99, 1.01] | 0.940 |
|  | Level | 1.02 [0.28, 3.72] | 0.974 |
|  | Post-slope | 0.95 [0.60, 1.51] | 0.843 |
| Ever smoker (n=8,746) | Time | 1.06 [0.90, 1.25] | 0.502 |
|  | Time^2^ | 1.00 [0.99, 1.01] | 0.905 |
|  | Level | 2.47 [0.33, 18.39] | 0.378 |
|  | Post-slope | 0.86 [0.52, 1.44] | 0.568 |

Note: where appropriate, models adjusted for sex and school year

Table 5: Annual prevalence and CI (95%) of regular e-cigarette use among pupils in Wales between 2015 and 2017, overall and by sex and school year

|  |  | 95% CI | |  | 95% CI | |  | 95% CI | |  | 95% CI | |  | 95% CI | |
| --- | --- | --- | --- | --- | --- | --- | --- | --- | --- | --- | --- | --- | --- | --- | --- |
| Year | **Total**  **(%)** | Low | High | **Boys**  **(%)** | Low | High | **Girls**  **(%)** | Low | High | **Year 9**  **(%)** | Low | High | **Year 11**  **(%)** | Low | High |
| 2015 | 4.2 | 3.9 | 4.6 | 5.1 | 4.5 | 5.7 | 3.5 | 3.0 | 4.0 | 2.8 | 2.4 | 3.2 | 6.0 | 5.3 | 6.7 |
| 2017 | 4.8 | 4.6 | 5.1 | 6.1 | 5.8 | 6.5 | 3.6 | 3.3 | 3.9 | 3.6 | 3.3 | 3.9 | 6.4 | 6.0 | 6.8 |

Table 6: Odds ratios (with 95% CIs) for regular use of e-cigarettes among pupils in Wales between 2015 and 2017 by ethnicity and socioeconomic status (before and after analyses)

|  |  |  | *P* |
| --- | --- | --- | --- |
| White  (n=42,246) | Time | 1.09 [1.00, 1.20] | 0.047 |
| BME  (n=4,291) | Time | 1.01 [0.82, 1.25] | 0.927 |
| High SES  (n=23,278) | Time | 1.13 [1.02, 1.25] | 0.019 |
| Low SES  (n=23,231) | Time | 1.08 [0.96, 1.21] | 0.211 |

Note: models adjusted for sex and school year

Table 7: Annual prevalence and CI (95%) of e-cigarette use (ever/regular) among pupils in England between 2014 and 2016, overall and by sex and age

|  |  |  | 95% CI | |  | 95% CI | |  | 95% CI | |  | 95% CI | |  | 95% CI | |
| --- | --- | --- | --- | --- | --- | --- | --- | --- | --- | --- | --- | --- | --- | --- | --- | --- |
|  | Year | **Total**  **(%)** | Low | High | **Boys**  **(%)** | Low | High | **Girls**  **(%)** | Low | High | **13 year**  **olds (%)** | Low | High | **15 year**  **olds (%)** | Low | High |
| Ever use | 2014 | 27.3 | 25.7 | 29.0 | 29.0 | 26.6 | 31.4 | 25.6 | 23.3 | 28.0 | 19.4 | 17.3 | 21.6 | 34.4 | 31.9 | 36.9 |
|  | 2016 | 32.5 | 31.3 | 33.8 | 35.2 | 33.4 | 37.1 | 30.1 | 28.4 | 31.8 | 23.8 | 22.2 | 25.4 | 41.2 | 39.3 | 43.0 |
| Regular use | 2014 | 1.7 | 1.2 | 2.2 | 1.9 | 1.3 | 2.8 | 1.4 | 0.8 | 2.2 | 1.0 | 0.5 | 1.7 | 2.3 | 1.6 | 3.2 |
|  | 2016 | 3.4 | 2.9 | 3.9 | 4.9 | 4.1 | 5.8 | 2.0 | 1.5 | 2.6 | 2.4 | 1.8 | 3.0 | 4.3 | 3.6 | 5.2 |

Table 8: Odds ratios (with 95% CIs) for primary and secondary outcomes among pupils in England between 2014 and 2016 by ethnicity

|  |  | Ever use of e-cigarettes | | Regular use of e-cigarettes | |
| --- | --- | --- | --- | --- | --- |
|  |  |  | *P* |  | *P* |
| White  (n=6,607) | Time | 1.16 [1.09, 1.23] | <0.001 | 1.47 [1.23, 1.77] | <0.001 |
| BME  (n=1,278) | Time | 1.14 [1.00, 1.30] | 0.044 | 1.38 [0.90, 2.11] | 0.138 |

Note: models adjusted for sex and age

Table 9: Prevalence and CI (95%) of ever energy drink use among pupils in Wales between November 2013 and December 2017, overall and by sex and school year

|  |  |  | 95% CI | |  | 95% CI | |  | 95% CI | |  | 95% CI | |  | 95% CI | |
| --- | --- | --- | --- | --- | --- | --- | --- | --- | --- | --- | --- | --- | --- | --- | --- | --- |
| Month-Year | Time | **Total**  **(%)** | Low | High | **Boys**  **(%)** | Low | High | **Girls**  **(%)** | Low | High | **Year 9**  **(%)** | Low | High | **Year 11**  **(%)** | Low | High |
| 11/2013 | 0 | 55.3 | 52.5 | 58.0 | 62.2 | 58.2 | 66.0 | 48.6 | 44.7 | 52.5 | 53.9 | 50.0 | 57.7 | 57.0 | 53.0 | 61.0 |
| 12/2013 | 1 | 52.7 | 49.5 | 55.8 | 60.2 | 55.8 | 64.4 | 44.4 | 39.9 | 49.1 | 52.2 | 47.8 | 56.5 | 53.5 | 48.8 | 58.1 |
| 01/2014 | 2 | 48.9 | 45.3 | 52.5 | 56.6 | 51.5 | 61.5 | 40.5 | 35.5 | 45.8 | 50.8 | 46.0 | 55.6 | 46.4 | 40.9 | 51.9 |
| 02/2014 | 3 | 50.2 | 45.5 | 54.9 | 59.7 | 53.2 | 65.9 | 38.9 | 32.2 | 46.0 | 49.2 | 43.0 | 55.4 | 51.6 | 44.2 | 59.0 |
| 03/2014 | 4 | 54.5 | 32.2 | 75.6 | 61.5 | 31.6 | 86.1 | 44.4 | 13.7 | 78.8 | - | - | - | 54.5 | 32.2 | 75.6 |
| 09/2015 | 22 | 54.1 | 51.6 | 56.6 | 59.9 | 56.1 | 63.7 | 49.8 | 46.4 | 53.1 | 56.9 | 53.5 | 60.3 | 50.8 | 47.0 | 54.5 |
| 10/2015 | 23 | 53.0 | 51.5 | 54.5 | 58.5 | 56.3 | 60.6 | 47.9 | 45.9 | 50.1 | 52.9 | 51.0 | 54.8 | 53.2 | 50.7 | 55.7 |
| 11/2015 | 24 | 51.6 | 50.2 | 52.9 | 57.5 | 55.6 | 59.4 | 46.2 | 44.4 | 48.0 | 53.6 | 51.7 | 55.5 | 49.5 | 47.7 | 51.4 |
| 09/2017 | 46 | 42.8 | 41.0 | 44.7 | 51.4 | 48.7 | 54.1 | 35.5 | 33.1 | 38.0 | 39.7 | 37.0 | 42.4 | 45.3 | 42.9 | 47.8 |
| 10/2017 | 47 | 44.7 | 43.7 | 45.7 | 51.6 | 50.1 | 53.0 | 37.9 | 36.5 | 39.3 | 44.7 | 43.4 | 46.0 | 44.7 | 43.1 | 46.2 |
| 11/2017 | 48 | 43.6 | 42.7 | 44.5 | 51.2 | 49.9 | 52.5 | 35.8 | 34.5 | 37.0 | 43.7 | 42.5 | 44.9 | 43.4 | 41.9 | 44.8 |
| 12/2017 | 49 | 41.7 | 40.9 | 42.6 | 49.6 | 48.4 | 50.8 | 34.2 | 33.1 | 35.3 | 41.0 | 39.9 | 42.1 | 42.6 | 41.4 | 43.8 |

Table 10: Odds ratios (with 95% CIs) for ever use of energy drinks among pupils in Wales between 2013 and 2017 by ethnicity and socioeconomic status (segmented regression analyses)

|  |  | Linear models | *P* | Quadratic models | *P* |
| --- | --- | --- | --- | --- | --- |
| White  (n=47,137) | Time (month-year) | 1.00 [0.99, 1.01] | 0.936 | 0.99 [0.92, 1.07] | 0.825 |
|  | Time^2^ | - | - | 1.00 [1.00, 1.00] | 0.828 |
|  | Level | 1.38 [0.60, 3.21] | 0.449 | 1.45 [0.57, 3.64] | 0.435 |
|  | Post-slope | 0.96 [0.92, 1.01] | 0.112 | 0.94 [0.75, 1.18] | 0.590 |
| BME  (n=4,787) | Time | 1.00 [0.99, 1.01] | 0.807 | 1.08 [0.93, 1.26] | 0.317 |
|  | Time^2^ | - | - | 1.00 [0.99, 1.00] | 0.324 |
|  | Level | 2.54 [0.48, 13.39] | 0.273 | 1.75 [0.26, 11.60] | 0.564 |
|  | Post-slope | 0.92 [0.84, 1.01] | 0.067 | 1.14 [0.73, 1.79] | 0.568 |
| High SES  (n=25,644) | Time | 1.00 [0.99, 1.01] | 0.993 | 1.01 [0.92, 1.11] | 0.783 |
|  | Time^2^ | - | - | 1.00 [1.00, 1.00] | 0.782 |
|  | Level | 1.53 [0.56, 4.17] | 0.401 | 1.44 [0.47, 4.38] | 0.524 |
|  | Post-slope | 0.96 [0.90, 1.01] | 0.116 | 0.99 [0.75, 1.31] | 0.963 |
| Low SES  (n=25,836) | Time | 1.00 [0.99, 1.01] | 0.772 | 0.98 [0.90, 1.07] | 0.596 |
|  | Time^2^ | - | - | 1.00 [1.00, 1.00] | 0.609 |
|  | Level | 1.31 [0.54, 3.16] | 0.550 | 1.46 [0.56, 3.82] | 0.435 |
|  | Post-slope | 0.97 [0.92, 1.01] | 0.172 | 0.91 [0.70, 1.17] | 0.441 |

Note: models adjusted for sex and school year

Table 11: Odds ratios (and 95% CIs) for ever use of energy drinks among pupils in Wales between 2013 and 2017, overall and by sex, school year, and smoking status – quadratic models (segmented regression analyses)

|  |  |  | P |
| --- | --- | --- | --- |
| All  (n=52,794) | Time (month-year) | 1.00 [0.93, 1.08] | 0.937 |
|  | Time^2^ | 1.00 [1.00, 1.00] | 0.947 |
|  | Level | 1.36 [0.64, 3.40] | 0.510 |
|  | Post-slope | 0.97 [0.77, 1.21] | 0.772 |
| Boys (n=25,888) | Time | 1.00 [0.92, 1.10] | 0.923 |
|  | Time^2^ | 1.00 [1.00, 1.00] | 0.858 |
|  | Level | 1.26 [0.46, 3.44] | 0.647 |
|  | Post-slope | 1.00 [0.76, 1.30] | 0.982 |
| Girls (n=26,906) | Time | 1.00 [0.91, 1.10] | 0.997 |
|  | Time^2^ | 1.00 [1.00, 1.00] | 0.925 |
|  | Level | 1.49 [0.49, 4.53] | 0.479 |
|  | Post-slope | 0.94 [0.71, 1.23] | 0.633 |
| Year 9  (n=29,545) | Time | 1.01 [0.92, 1.11] | 0.853 |
|  | Time^2^ | 1.00 [1.00, 1.00] | 0.911 |
|  | Level | 1.22 [0.38, 3.96] | 0.742 |
|  | Post-slope | 0.97 [0.73, 1.30] | 0.855 |
| Year 11  (n=23,249) | Time | 0.99 [0.91, 1.08] | 0.807 |
|  | Time^2^ | 1.00 [1.00, 1.00] | 0.867 |
|  | Level | 1.58 [0.55, 4.55] | 0.394 |
|  | Post-slope | 0.94 [0.74, 1.20] | 0.637 |
| Never smoker  (n=41,457) | Time | 0.99 [0.91, 1.07] | 0.770 |
|  | Time^2^ | 1.00 [1.00, 1.00] | 0.727 |
|  | Level | 1.51 [0.58, 3.94] | 0.403 |
|  | Post-slope | 0.92 [0.72, 1.16] | 0.468 |
| Ever smoker  (n=8,805) | Time | 1.12 [0.98, 1.27] | 0.087 |
|  | Time^2^ | 1.00 [0.99, 1.00] | 0.092 |
|  | Level | 1.28 [0.33, 5.01] | 0.725 |
|  | Post-slope | 1.30 [0.89, 1.89] | 0.180 |

Note: where appropriate, models adjusted for sex and/or school year

Table 12: Odds ratios (with 95% CIs) for ever use of energy drinks among pupils in Wales between 2013 and 2017 with smoking status (ever smoked = 1) as a modelled covariate (segmented regression analyses)

|  |  | Linear models | *P* | Quadratic models | *P* |
| --- | --- | --- | --- | --- | --- |
| All  (n=50,262) | Time (month-year) | 1.00 [1.00, 1.01] | 0.428 | 1.01 [0.94, 1.09] | 0.784 |
|  | Time^2^ | - | - | 1.00 [1.00, 1.00] | 0.828 |
|  | Level | 1.49 [0.66, 3.36] | 0.332 | 1.43 [0.58, 3.56] | 0.437 |
|  | Post-slope | 0.95 [0.91, 1.00] | 0.033 | 0.97 [0.78, 1.22] | 0.821 |
|  | Smoking status | 3.67 [3.44, 3.91] | <0.001 | 3.67 [3.44, 3.91] | <0.001 |
| Boys  (n=24,492) | Time (month-year) | 1.00 [0.99, 1.00] | 0.451 | 1.00 [0.91, 1.10] | 0.991 |
|  | Time^2^ | - | - | 1.00 [1.00, 1.00] | 0.965 |
|  | Level | 1.32 [0.56, 3.12] | 0.528 | 1.31 [0.48, 3.56] | 0.603 |
|  | Post-slope | 0.97 [0.93, 1.02] | 0.214 | 0.98 [0.74, 1.28] | 0.863 |
|  | Smoking status | 3.39 [3.11, 3.69] | <0.001 | 3.39 [3.11, 3.69] | <0.001 |
| Girls  (n=25,770) | Time (month-year) | 1.01 [1.00, 1.01] | 0.050 | 1.02 [0.93, 1.11] | 0.683 |
|  | Time^2^ | - | - | 1.00 [1.00, 1.00] | 0.798 |
|  | Level | 1.74 [0.62, 4.91] | 0.294 | 1.64 [0.54, 5.05] | 0.385 |
|  | Post-slope | 0.93 [0.88, 0.99] | 0.021 | 0.96 [0.74, 1.26] | 0.791 |
|  | Smoking status | 3.98 [3.68, 4.30] | <0.001 | 3.98 [3.68, 4.31] | <0.001 |
| Year 9  (n=28,071) | Time (month-year) | 1.01 [1.00, 1.01] | 0.092 | 1.00 [0.91, 1.10] | 0.993 |
|  | Time^2^ | - | - | 1.00 [1.00, 1.00] | 0.903 |
|  | Level | 1.42 [0.51, 3.95] | 0.507 | 1.46 [0.46, 4.59] | 0.520 |
|  | Post-slope | 0.95 [0.89, 1.00] | 0.062 | 0.93 [0.71, 1.23] | 0.613 |
|  | Smoking status | 5.60 [5.08, 6.18] | <0.001 | 5.60 [5.08, 6.18] | <0.001 |
| Year 11  (n=22,191) | Time (month-year) | 1.00 [0.99, 1.01] | 0.692 | 1.01 [0.93, 1.10] | 0.793 |
|  | Time^2^ | - | - | 1.00 [1.00, 1.00] | 0.765 |
|  | Level | 1.60 [0.60, 4.25] | 0.342 | 1.51 [0.53, 4.31] | 0.445 |
|  | Post-slope | 0.96 [0.91, 1.01] | 0.116 | 0.99 [0.78, 1.26] | 0.953 |
|  | Smoking status | 3.01 [2.78, 3.25] | <0.001 | 3.01 [2.79, 3.25] | <0.001 |

Note: where appropriate, models adjusted for sex and school year

**Interaction terms**

Table 12: Odds ratios for interaction terms for all outcomes among pupils in Wales

| Interaction terms | Ever use of  e-cigarettes  (n=51,056)^a^ | *P* | Ever use of  energy drinks  (n=52,794)^a^ | *P* | Regular use of e-cigarettes (n=47,318)^b^ | *P* |
| --- | --- | --- | --- | --- | --- | --- |
| Time * sex | 1.00 [0.99, 1.00] | 0.029 | 1.00 [0.99, 1.00] | 0.034 | 0.92 [0.79, 1.06] | 0.057 |
| Level * sex | 0.85 [0.75, 0.96] | 0.007 | 0.87 [0.79, 0.95] | 0.003 | **-** | **-** |
| Trend * sex | 0.99 [0.98, 1.00] | 0.015 | 0.99 [0.99, 1.00] | 0.003 | - | - |
| Time * school year | 1.00 [1.00, 1.01] | 0.612 | 1.00 [1.00, 1.01] | 0.169 | 0.91 [0.79, 1.05] | 0.204 |
| Level * school year | 1.02 [0.88, 1.19] | 0.760 | 1.11 [1.01, 1.23] | 0.035 | **-** | **-** |
| Trend * school year | 1.00 [0.99, 1.01] | 0.693 | 1.01 [1.00, 1.01] | 0.034 | - | - |

^a^ Based on segmented regression models (linear); ^b^ Based on before and after models

Table 13: Odds ratios for interaction terms (sex and age) for all outcomes among pupils in England (before and after analysis)

| Interaction terms | Ever use of  e-cigarettes  (n=8,178) | *P* | Regular use of  e-cigarettes  (n=8,178) | *P* |
| --- | --- | --- | --- | --- |
| Time * sex | 0.95 [0.86, 1.05] | 0.314 | 0.72 [0.51, 1.00] | 0.053 |
| Time * age | 1.02 [0.92, 1.13] | 0.713 | 0.92 [0.64, 1.32] | 0.646 |

**Process indicators (Wales only)**

Pupil responses to questions from the 2017 SHRN/HBSC survey on e-cigarettes and associated processes, asked of a subsample of whole SHRN/HBSC sample. Frequencies relate to pupils in years 9 and 11 only. Rounding errors mean percentages may not always sum correctly. Overall sample sizes will differ due to some questions not being asked of all respondents.

Table 14: Health risk perceptions for tobacco and e-cigarette use, and content of last e-cigarette used, among 13 and 15 year olds in Wales in 2017 by ethnicity and socioeconomic status

|  | Perceived harms of smoking | | | | Nicotine content of last e-cigarette used  (ever users only) | | | | |
| --- | --- | --- | --- | --- | --- | --- | --- | --- | --- |
|  | Smoking worse  (%) | Vaping worse | Equally harmful | Don’t know | Nicotine | Flavour / water vapour only | Cannabis or cannabis oil | Something else | Don’t know |
| White | 5,560 (48.3) | 347 (3.0) | 4,058 (35.2) | 1,559 (13.5) | 2,418 (30.2) | 4,228 (52.7) | 201  (2.5) | 69  (0.9) | 1,105 (13.8) |
| BME | 500 (47.0) | 29  (2.7) | 389 (36.5) | 147 (13.8) | 212 (27.8) | 405 (53.0) | 41  (5.4) | 9  (1.2) | 97 (12.7) |
| High SES | 3,301 (49.6) | 159 (2.4) | 2,329 (35.0) | 867 (13.0) | 1,241 (28.5) | 2,361 (54.2) | 134  (3.1) | 44  (1.0) | 573 (13.2) |
| Low SES | 2,831 (45.9) | 229 (3.7) | 2,200 (35.7) | 908 (14.7) | 1,418 (31.3) | 2,320 (51.2) | 113  (2.5) | 36  (0.8) | 647 (14.3) |

Table 15: Perceptions of parental attitudes to regulation of smoking and e-cigarette use among 13 and 15 year olds in Wales in 2017 by ethnicity and socioeconomic status

|  |  | Try to stop me  (%) | Try persuading me to stop | Do nothing | Encourage me to vape |
| --- | --- | --- | --- | --- | --- |
| E-cigarettes (non-users) | White  *(n=10,106; 91.8%)* | 7,449  (74.2) | 2,228  (22.1) | 315  (3.1) | 64  (0.6) |
|  | BME  *(n=907; 8.2%)* | 717  (79.1) | 156  (17.2) | 26  (2.9) | 8  (0.9) |
|  | High SES  *(n=5,875; 52.5%)* | 4,521  (77.0) | 1,186  (20.2) | 135  (2.3) | 33  (0.6) |
|  | Low SES  *(n=5,322; 47.5%)* | 3,844  (72.2) | 1,228  (23.1) | 210  (4.0) | 40  (0.8) |
| Smoking (non-smokers) | White  *(n=10,310; 91.7%)* | 8,780  (85.2) | 1,373  (13.2) | 121  (1.2) | 36  (0.4) |
|  | BME  *(n=937; 8.3%)* | 827  (88.3) | 97  (10.4) | 9  (1.0) | 4  (0.4) |
|  | High SES  *(n=6,019; 52.6%)* | 5,238  (87.0) | 712  (11.8) | 55  (0.9) | 14  (0.2) |
|  | Low SES  *(n=5,423; 47.4%)* | 4,536  (83.6) | 783  (14.4) | 78  (1.4) | 26  (0.5) |

Table 16: Modes of obtaining e-cigarettes among 13 and 15 year olds in Wales in 2017 by ethnicity and socioeconomic status

|  | Buy from shop (%) | Internet | From adults | From peers | Take | From siblings | Other |
| --- | --- | --- | --- | --- | --- | --- | --- |
| White  *(n=2,934; 100%)* | 417  (14.2) | 188  (6.4) | 487  (16.6) | 962  (32.8) | 229  (7.8) | 98  (0.3) | 576  (19.6) |
| BME  *(n=341; 100%)* | 82  (24.1) | 35  (10.3) | 60  (17.6) | 77  (22.6) | 50  (14.7) | 19  (0.6) | 73  (21.4) |
| High SES  *(n=1,619; 100%)* | 285  (17.6) | 106  (6.6) | 284  (17.5) | 506  (31.3) | 131  (8.1) | 60  (0.3) | 334  (20.6) |
| Low SES  *(n=1,694; 100%)* | 220  (13.0) | 121  (7.1) | 269  (15.9) | 543  (32.1) | 154  (9.1) | 59  (0.3) | 320  (18.9) |

Note: multiple responses allowed. Figures therefore reflect numbers of pupils that acquired e-cigarettes via each method (and as a percentage of the total sample). Current e-cigarette users only.

Table 17: Prevalence and locations of exposure to e-cigarette marketing among 13 and 15 year olds in Wales in 2017 by ethnicity and socioeconomic status

|  | Bus shelter (%) | Side of bus | Billboard | Supermarket, petrol station, newsagent, vape shop | Internet | Phone box | Other | No exposure |
| --- | --- | --- | --- | --- | --- | --- | --- | --- |
| White  *(n=12,057; 91.3%)* | 2,516  (20.9) | 1,323  (11.0) | 1,754  (14.6) | 5,103  (42.2) | 4,786  (39.7) | 940  (7.8) | 2,246  (18.6) | 4,011  (33.3) |
| BME  *(n=1,154; 8.7%)* | 232  (20.1) | 146  (12.7) | 208  (18.0) | 421  (36.5) | 403  (34.9) | 109  (9.5) | 218  (18.9) | 380  (32.9) |
| High SES  *(n=6,978; 51.7%)* | 1,562  (22.4) | 848  (12.2) | 1,158  (16.6) | 3,064  (43.9) | 2,853  (40.9) | 619  (8.9) | 1,395  (20.0) | 2,206  (31.6) |
| Low SES  *(n=6,525; 48.3%)* | 1,231  (18.9) | 644  (9.9) | 839  (12.9) | 2,540  (38.9) | 2,408  (36.9) | 448  (6.9) | 1,114  (17.1) | 2,288  (35.1) |

Note: multiple responses allowed. Figures therefore reflect numbers of pupils exposed to e-cigarette marketing via each mechanism (and as a percentage of the total sample)
